# Implementation of a national rheumatic fever and rheumatic heart disease prevention and control program in Egypt: rationale and objectives

**DOI:** 10.3389/fcvm.2026.1833934

**Published:** 2026-09-21

**Authors:** Susy Kotit, Wagida Anwar, Alyaa Kotby, Sameh Shaheen, Sahar S. Sheta, Salah Zaher, Mohamed Abdel-Hady, Ahmed Ibrahim Nassar, Soleiman Gharib Mahdy, Mohamed Magdy Abouelkheir, Ragab Mahfouz, Adel Hamdy Mahmoud, Yasser Abdelhady, Laila Shalaby, Mona Khalifa, Mohamed Hassany, Alaa Elghamrawy, Fathi Maklady

**Affiliations:** 1 Aswan Heart Centre, Magdi Yacoub Heart Foundation, Cairo, Egypt; 2 Ain Shams University, Cairo, Egypt; 3 Kasr el Aini, Cairo University, Cairo, Egypt; 4 Alexandria University, Alexandria, Egypt; 5 National Heart Institute, Cairo, Egypt; 6 Mansoura University, Mansoura, Egypt; 7 Zagazig University, Zagazig, Egypt; 8 Minia University, Minia, Egypt; 9 Beni Suef University, Beni Suef, Egypt; 10 General Management of Health Initiatives, Cairo, Egypt; 11 Ministry of Health and Population, Cairo, Egypt; 12 Mahalla Cardiac Center, Mahalla, Egypt; 13 Suez Canal University, Ismailia, Egypt

**Keywords:** acute rheumatic fever, echocardiography screening, Egypt, prevention, prophylaxis, registries, rheumatic heart disease

## Abstract

**Background:**

Rheumatic heart disease (RHD) remains a major public health challenge in Egypt, with a school-age prevalence of 0.9%–2.5%. Despite national initiatives since 2006, control has been constrained by low public and provider awareness, suboptimal secondary prophylaxis, misdiagnosis, and fragmented surveillance. This report outlines the rationale and structure of Egypt's national RF/RHD prevention and control program, which integrates screening, digital surveillance, capacity building, and research to support scalable RHD control in endemic settings.

**Methods:**

The program comprises (1) mandatory RF notification and a national electronic registry for public-sector cases and screening positives; (2) nationwide school-based echocardiographic screening of >10,000 children across 27 governorates using standardized clinical examination and World Heart Federation criteria; (3) Characterization of regional patterns and determinants of RHD; (4) Development of region-specific risk stratification tools, guidelines, diagnostic criteria and prevention strategies; (5) nationwide multimedia awareness campaigns; (6) Capacity building through continuous medical education and training at all care levels; and (7) and embedded high-impact research and clinical trials.

**Conclusions:**

Egypt's national RF/RHD prevention and control program provides a comprehensive and scalable framework that combines school-based screening, mandatory notification, a national registry, workforce training, public awareness, and embedded research to improve case detection, support timely prophylaxis, and generate local evidence for risk stratification and policy, modelling an approach suitable for other endemic settings.

## Introduction

Rheumatic heart disease (RHD), resulting from Rheumatic Fever (RF) following untreated Group A streptococcal (GAS) infection, remains a major public health challenge in many low- and middle-income countries, causing substantial cardiovascular morbidity and premature mortality, with largely preventable clinical, economic, and societal consequences.

In Egypt, RHD continues to contribute significantly to cardiovascular disease burden, particularly among children, adolescents, young adults and women during their most productive years (1–3). School-based echocardiographic screenings across several governorates report prevalence rates ranging 0.9% to 2.5% (4–8), while subclinical carditis is identified in approximately 30% of acute RF cases (9).

Key national risk factors include genetic predisposition (10, 11), female gender (1, 3, 12, 13), rural residence (3, 14), overcrowding, chronic tonsillitis (4, 12, 15), low socioeconomic status (12, 15), and family history of RF/RHD (15), with notable regional variation leading to heterogeneous disease patterns and outcomes.

Awareness remains limited at both community and healthcare levels. Public understanding of the link between sore throat and RHD is limited (16), and caregivers often lack knowledge of RF symptoms, complications, and appropriate management (14). Physician knowledge gaps and variability in clinical practice (17), contribute to substantial misdiagnosis at the primary-care level (∼42%) (12, 18).

Adherence to benzathine penicillin prophylaxis is also suboptimal (∼31%) (1, 3, 12, 15), resulting in recurrent RF episodes and accelerated disease progression.

Consequently, RHD leads to recurrent hospitalizations, heart failure, stroke, pregnancy—related complications, and premature mortality, often requiring costly surgical and interventional care (1, 2, 19, 20). This places sustained pressure on families, healthcare systems, and national productivity, particularly in resource—constrained settings.

In response, the Egyptian Ministry of Health and Population established a National RF/RHD Prevention and Control Program committee in 2006 (3, 12). This resulted in the assessment of 25,117 children at El-Mahalla RHD center from 2006 to 2023 in which participants completed a questionnaire and underwent ECG and echocardiographic assessment. The majority of the screened children were aged 5–15 years (62.0%), female (61.2%), and rural residents (61.5%), and 49.0% had low socioeconomic status. RHD was diagnosed in 12% of them, and among 21% of their siblings and relatives (18).

Building on these efforts, the 2025 RF/RHD national committee has advanced a more integrated strategy combining public-sector electronic registry infrastructure, echocardiographic school-based screening, standardized care pathways, strengthened secondary prophylaxis, capacity building, public awareness, and development of research-driven policy.

This report outlines the rationale and objectives of the 2025–2027 program phase in alignment with national and global priorities (21–23), with emphasis on equity and scalability for other RHD—endemic settings.

### Rationale of Egypt's 2025 RF/RHD national program

Persistent system—level gaps, including fragmented surveillance, delayed diagnosis, inconsistent delivery of secondary prophylaxis, and limited continuity of follow-up, continue to sustain RF transmission and progression to advanced valvular disease. These challenges are compounded by the absence of a unified national data infrastructure, which limits accurate burden estimation (12), longitudinal outcome tracking, and evidence-based policymaking, thereby contributing to underrecognition of high-risk populations and widening regional inequities. A coordinated health-system response is therefore required.

A centralized national electronic RF/RHD registry with mandatory RF notification is therefore essential to enable systematic case identification, standardized data-capture, and real-time monitoring of disease progression, treatment adherence, and outcomes across diverse geographic settings. Such a registry also enables characterization of regional heterogeneity and generates real-world evidence to inform risk stratification, resource allocation, and policy decisions.

Echocardiographic screening plays a critical role in this strategy by enhancing the detection of subclinical carditis and early RHD compared with clinical examination alone. In endemic settings, school-based programs offer a scalable platform for identifying cases before irreversible valvular damage develops, allowing timely initiation of prophylaxis, structured follow-up, and referral. Linking screening to a registry-based framework ensures that early detection translates into sustained longitudinal care and improved outcomes, while enabling evaluation of disease trajectories. This is particularly relevant in Egypt, where RHD epidemiology is highly heterogeneous, reflecting variations in socioeconomic conditions, healthcare access, streptococcal exposure, diagnostic capacity, and adherence to prophylaxis which collectively drive disparities in disease burden and outcomes.

Strengthening workforce capacity through standardized training and continuous medical education (CME) is essential to improve diagnostic accuracy and referral pathways, ensuring consistency of care. Public awareness initiatives are important to enhance early recognition of streptococcal infection and reinforce adherence to prophylaxis.

Dedicated RF/RHD units within public hospitals and linked provincial centers will support continuity of care, quality improvement, and long-term follow-up while serving as hubs for expertise and supervision. Embedding a research and clinical trial platform will enable real-world evaluation of disease progression, prognostic factors, and delivery models, accelerating translation of evidence into practice.

Together, these components form an integrated national strategy that combines screening, mandatory notification, centralized registry infrastructure, standardized care pathways, education, and research. This approach provides a scalable and sustainable framework to reduce disease progression, morbidity and the need for surgical intervention, while improving equity in cardiovascular outcomes and serving as a model for endemic settings.

## Aims and objectives

The detailed aims and objectives of Egypt's 2025 RF/RHD National Program are described in Figure 1.

**Figure 1:**
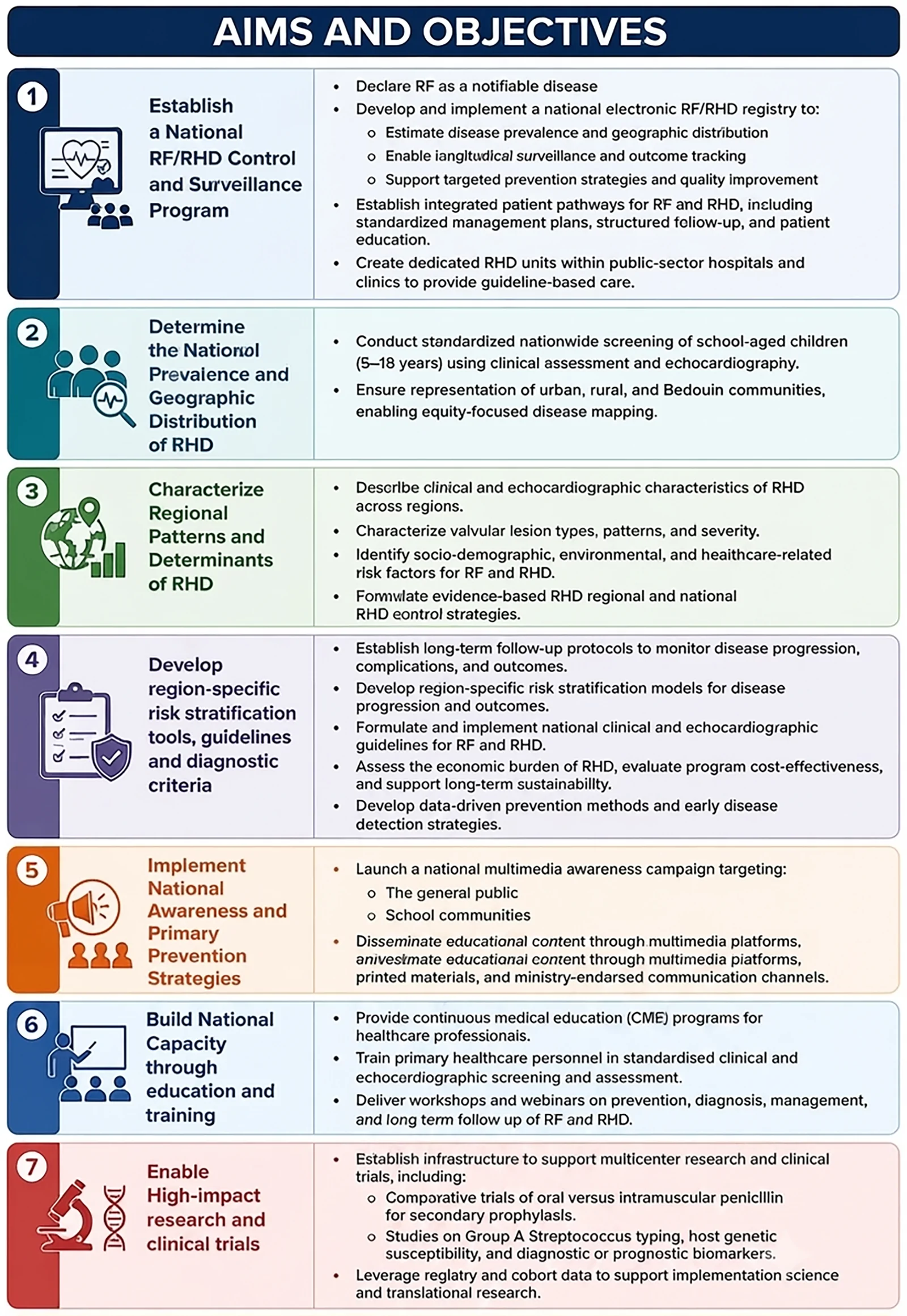
Egypt's 2025 RF/RHD national program aims and objectives.

### Phased implementation framework

To ensure feasibility, scalability, and sustainability, the program will be implemented through a phased, stepwise approach**,** allowing early system building, progressive expansion, and continuous refinement based on generated evidence. The detailed timeline is shown in Figure 2.

**Figure 2:**
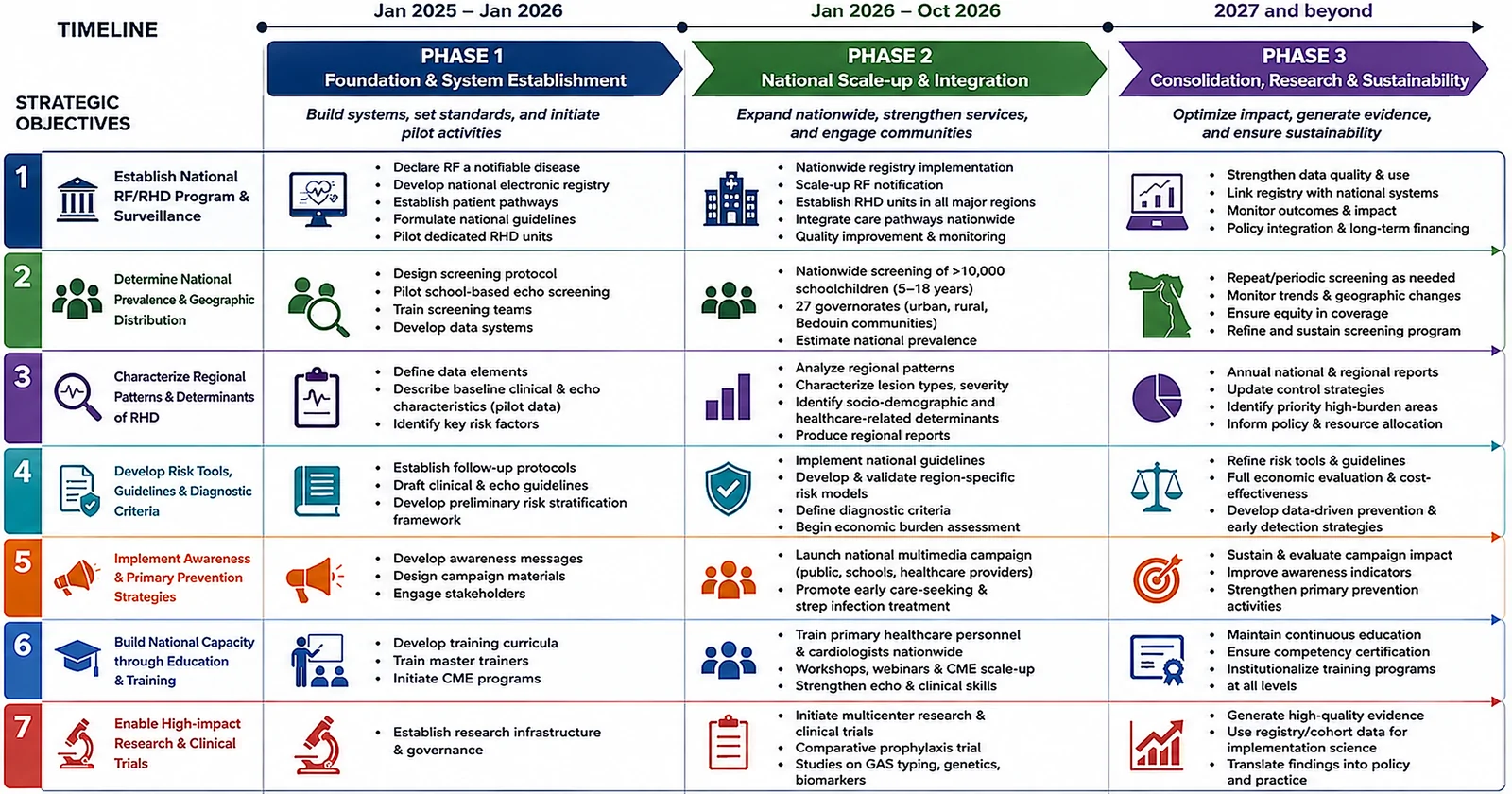
Timeline of the phased framework of Egypt's 2025 RF/RHD national program.

## Methods

This study describes the design and implementation of Egypt's National Acute Rheumatic Fever (RF)/Rheumatic Heart Disease (RHD) Prevention and Control Program (2025–2027), integrating mandatory RF notification, a national electronic registry, cross-sectional echocardiographic screening of >10,000 schoolchildren across 27 governorates, region-specific care pathways, continuous medical education, multimedia awareness campaigns, and a nested randomized clinical trial evaluating penicillin prophylaxis strategies.

### RF notification

Notification will be implemented through Ministry of Health and Population surveillance mechanisms. All individuals presenting at a public health facility with any stage of RF/RHD will be registered.

### RF/RHD national registry

A national electronic RF/RHD registry for clinical and echocardiographic data will track all cases nationwide, enabling targeted prevention, longitudinal surveillance, resource allocation, evidence-based interventions, and improved care.

All patients diagnosed with RF or RHD presenting in the public sector or at designated RHD centres will be enrolled, capturing detailed clinical, demographic, and treatment data at initial presentation and follow-up visits. Additionally, all children identified with RF or RHD through school-based echocardiographic screening will be registered, linking screening results to longitudinal care pathways. This dual-enrolment approach ensures complete case ascertainment, facilitates real-time progression tracking, supports clinical trials (e.g., prophylaxis optimization), and generates data for national reporting and guideline refinement.

The registry will collect the following data:

- **Demographic data:** date of birth, age at enrollment, sex, education level, address, contact information, governorate and school codes
- **Socioeconomic indicators:** household crowding (number of family members and rooms) and residential setting (urban/rural)
- **Clinical history:** history of tonsillitis, acute rheumatic fever, family history of RHD, and current symptoms (e.g., dyspnea, fatigue, palpitations)
- **Penicillin prophylaxis:** initiation date, regimen, and adherence
- **Intervention history:** prior surgical or percutaneous procedures
- **Medication:** current and prior medication use
- **Clinical examination findings:** presence and type of cardiac murmurs and other relevant physical findings
- **Echocardiographic assessment:** valve involvement (mitral and aortic), lesion type and severity (regurgitation/stenosis), Doppler measurements, and morphological features (WHF diagnostic criteria)
- **Diagnosis:** RHD classification
- **Plan:** referral pathway and follow-up schedule

### Data collection and management

All demographic, clinical, and echocardiographic data will be centrally managed and registered via secure, web-based software, with real-time data audits and regular quality assurance.

The national RF/RHD registry will serve as a centralized digital Electronic Data Capture (EDCS) platform linked with the Ministry of Health's existing electronic infrastructure, to track all cases nationwide, enabling longitudinal monitoring, resource allocation, evidence-based interventions follow-up and notifications.

Echocardiographic studies will be stored for re-evaluation and follow-up purposes in Picture Archiving and Communication System (PACS).

### School screening

Cross-sectional screening (stratified random sampling) with prospective registry and follow-up across all 27 Egyptian governorates, targeting schoolchildren aged 5–18 years. Schools will be stratified by urban, rural, and Bedouin regions to ensure representativeness. ​​

### Population and sampling

The program targets the screening of >10,000 schoolchildren aged 5–18 years, calculated based on an expected RHD prevalence of 1.5%, a precision of ±0.2%, and a significance level of *α* = 0.05, to achieve approximately 99% confidence in estimating national prevalence. To ensure representativeness, the sample is stratified by geography (urban vs. rural), as well as by age group and gender.

A multistage stratified cluster sampling approach is employed. Governorates are first stratified into urban and rural strata, followed by enumeration of schools within each stratum. Schools are then randomly selected using computer-generated algorithms, with proportional allocation according to population size, educational stage, and gender distribution. Within selected schools, students are randomly sampled using automated software to preserve demographic proportionality.

### Screening procedures

Every student in the selected sample will undergo comprehensive clinical examination and echocardiographic assessment following this standardized protocol:

- Detailed history: demographic data, prior rheumatic fever history, and current symptoms directly from the student.
- Cardiac examination: Trained physicians will follow a prespecified checklist, including inspection and palpation of the apex and pulsations; auscultation of the precordial region for S1/S2 and additional sounds; and reporting of any murmurs, with etiology differentiated via echocardiography and correlated with history.
- Echocardiographic assessment: On-site imaging using a portable echo machine for standard views. Diagnosis will be made according to the updated WHF RHD echocardiographic diagnostic criteria (24).

All echocardiography studies will undergo independent review by a panel of certified echocardiography consultants blinded to clinical findings to confirm the diagnosis, quality assurance, and reduce inter-observer variability.

### Patient pathways

RF/RHD units will be established within public-sector hospitals and clinics for the registration of RHD patients. Clear integrated pathways, including referral to specialized RHD clinics, and management plans have been formulated for the public sector.

Individuals with suspected RF or RHD at the time of screening will be directed to specialized RHD centres for further assessment, management and follow-up.

Contact points, information materials, and structured follow-up booklets will be provided to positive screening cases and patients with all forms of RHD.

Currently, 30 specialized RHD centers are established across different governorates. These centers function as hubs of expertise, providing comprehensive, disease-specific care, including early diagnosis, advanced imaging, standardized management protocols, medical treatment and penicillin prophylaxis, patient counselling, and structured long-term follow-up supported by registries. These specialized centers are intended to strengthen the public sector by facilitating early diagnosis, improvement of quality of care and adherence to treatment and follow-up. In addition, they play a critical role in supporting training, research, and policy development to optimize outcomes across the continuum of RHD care.

#### Continuous medical education (CME) and training

A total of 5000 primary health care personnel and cardiologists are to be trained annually through echocardiography programs and/or workshops and webinars.

Echocardiography training of primary health care personnel in standardized clinical/echo assessment for screening purposes follows structured programs, with hands-on training, final training examination and certifications through the Egyptian Society of Cardiology. Workshops and webinars on prevention, management, and follow-up of RF/RHD are to be provided through dedicated teaching centres and the ministry platforms and will be evaluated using pre- and post-CME surveys to assess changes in knowledge, confidence, and learning outcomes.

These interventions directly address documented drivers of disease progression in Egypt, including misdiagnosis, delayed presentation, variations in diagnostic criteria and management and poor adherence to secondary prophylaxis.

#### National multimedia awareness and primary prevention campaign

A national multimedia awareness and primary prevention campaign targeting the public, healthcare providers, and communities has been launched through ministry-endorsed information outlets.

Information material was developed targeting the public, healthcare providers, and communities to disseminate awareness via multimedia campaigns, printed materials, and ministry-endorsed information outlets.

#### Clinical trial

A nested randomized controlled trial will compare oral vs. intramuscular benzathine penicillin G prophylaxis for secondary prevention of RF recurrences and RHD progression, with prospective monitoring of adherence, clinical outcomes, and safety endpoints. The protocol, methodological framework, and logistical aspects of the clinical trial have not yet been established and will be addressed in a subsequent phase of the program.

## Ethics

Given that this initiative is a national, Ministry-led public health screening program, individual written parental consent is not required. Population-based screening programs, such as school-based health screening, are widely recognized as essential public health interventions, and ethically justify a waiver of parental permission, particularly when the intervention poses minimal risk, ensures confidentiality, and provides direct potential benefit through early detection and referral.

Approval for this program was granted by the Egyptian Ministry of Health and Population and the Ministry of Education for screening purposes, ensuring that participation remains voluntary, non-coercive, and conducted within a structured public health framework. All children are eligible for screening. Participants and their families are appropriately informed, and confidentiality is strictly maintained. Data collected is used exclusively for referral and clinical follow-up in line with the objectives of the program. Importantly, appropriate care pathways were ensured for children with positive screening results.

## Data analysis

Prospective descriptive and comparative statistics (with chi-square tests and odds ratios) will be performed to analyze prevalence, patterns, and risk factors. Economic analysis and outcome evaluation will follow.

Screening data will initially be summarized using weighted prevalence estimates with 95% confidence intervals accounting for the multistage clustered sampling design. Further analytic methods for registry-based longitudinal outcomes, risk stratification, and comparative analyses will be specified and reported in subsequent publications.

## Strategic objectives

The strategic objectives, key performance indicators and 3-year targets are listed in Figure 3.

**Figure 3:**
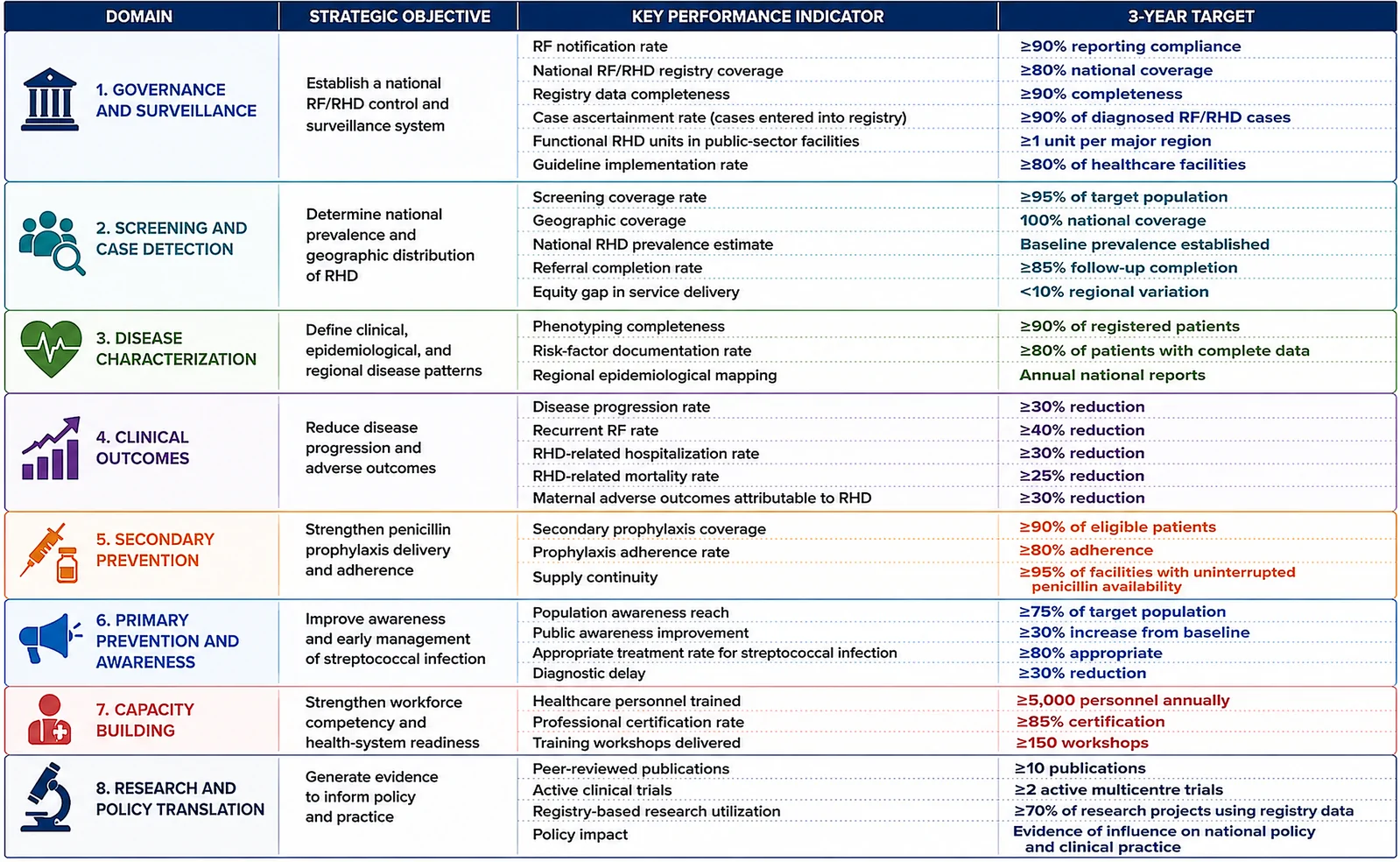
Strategic objectives, key performance indicators, and 3-year targets for Egypt's 2025–2027 RF/RHD national program.

### Progress to date

Until April 2026, a total of 3,317 students were screened across nine governorates. Screening activities are expected to resume after the national examination period and at the start of the new academic year. Echocardiographic studies are currently undergoing re-evaluation, with final statistical analysis to be conducted upon completion of the screening phase.

In parallel, 30 specialized RHD units have been established and equipped with the necessary infrastructure and trained personnel. Implementation of a nationwide registry within the public sector is currently pending.

Materials for a national multimedia awareness campaign have been developed, and training of primary healthcare personnel and cardiologists is ongoing, as well as the evaluation of training and pre- and post-CME surveys.

Continuous monitoring and evaluation of program objectives and progress will follow.

## Discussion

Egypt's national RF/RHD control program addresses persistent deficiencies in early detection, surveillance, secondary prevention, and continuity of care through an integrated, system-level strategy. By combining mandatory notification, a national electronic registry, echocardiographic screening, workforce capacity building (including CME and training), public awareness, and embedded research, the program shifts from fragmented, episodic interventions to a coordinated, evidence-driven and sustainable, system-integrated, national response. The initiative further introduces dedicated RHD centers, expands school-based screening, and strengthens referral pathways to support long-term follow-up and care delivery. This model integrates service delivery and knowledge generation, enabling real-world evaluation of disease patterns, adherence, and outcomes. Importantly, integration of surveillance and service delivery supports continuous program refinement through data-informed decision-making, offering strong potential for adaptation and scalability in other high-burden settings.

However, several implementation and sustainability challenges must be carefully considered. Logistical constraints related to school schedules and examination periods may affect screening coverage. Mitigation strategies include offering flexible, community-based screening at designated RHD units to complement school-based programs.

Workforce turnover within the public sector represents another critical challenge, necessitating strict governance, continuous training and quality assurance efforts. This may be addressed through sub-specialization pathways in RHD care and retention strategies such as competency-based certification and career incentives to ensure workforce continuity.

Sustained community engagement is also essential to ensure uptake of screening and adherence to prophylaxis. Awareness campaigns should be continuous, culturally sensitive, and targeted toward families, schools and community leaders. Integration of health education into school curricula and partnership with community networks may further strengthen engagement and sustainability.

In addition, long-term sustainability of the national electronic registry will depend on robust digital infrastructure, interoperability across healthcare levels, and consistent data governance, including data quality assurance, completeness, and protection of patient confidentiality. Standardized data collection protocols and periodic audits are required to address regional variability and ensure data quality and comparability.

Ensuring adherence to secondary prophylaxis and long-term follow-up represents another major challenge, particularly in underserved and highly mobile populations, where loss to follow-up, access barriers, and competing socioeconomic priorities may undermine program effectiveness. Strategies such as digital reminders, decentralized drug delivery, and stronger linkage with primary care services may improve adherence and continuity.

Financial sustainability is also a critical consideration. The scale-up and maintenance of screening programs, registry infrastructure, workforce training, and dedicated RHD services require sustained funding commitments, and may be vulnerable to competing health system priorities and economic constraints. Embedding RHD services within existing universal health coverage frameworks and leveraging public–private partnerships may support long-term financial resilience.

Health system heterogeneity across regions, including disparities in infrastructure, workforce availability, and access to diagnostic tools such as echocardiography, may lead to uneven implementation and impact, potentially perpetuating existing inequities if not carefully addressed. Targeted resource allocation and phased implementation strategies may be required to ensure equitable coverage.

Furthermore, integrating new care pathways into existing health system workflows may encounter resistance or operational challenges, requiring strong governance, stakeholder engagement, and continuous monitoring to ensure fidelity of implementation, supported by clear accountability structures and performance indicators.

Ultimately, the success of this model will depend on sustained financing, strong governance, effective intersectoral coordination and implementation across heterogeneous geographic and socioeconomic contexts, and continuous evaluation to ensure adaptability, efficiency, and equitable impact.

## Conclusion

Egypt's national RF/RHD control program provides a comprehensive and scalable framework, that integrates echocardiographic screening, mandatory notification, a centralized electronic registry, workforce capacity building, public awareness, and embedded research.

By linking early detection with longitudinal follow-up and data-driven decision-making, the program aims to improve case identification, optimize prophylaxis, and generate context-specific evidence to guide risk stratification and policy. This integrated model provides a practical and scalable approach that could serve as a blueprint for other endemic settings.

## Data Availability

The data and results supporting the rationale and implementation of this study are available in previously published articles and are cited throughout the text. Further inquiries can be directed to the corresponding author/s.
